# Vaccination prepartum enhances the beneficial effects of melatonin on the immune response and reduces platelet responsiveness in sheep

**DOI:** 10.1186/1746-6148-8-84

**Published:** 2012-06-20

**Authors:** Sergio Regodón, Asunción Ramos, María P Míguez, Antonio Carrillo-Vico, Juan A Rosado, Isaac Jardín

**Affiliations:** 1 Department of Animal Medicine, University of Extremadura, 10003, Cáceres, Spain; 2 Department of Animal Health, University of Extremadura, 10003, Cáceres, Spain; 3 Deparment of Medical Biochemistry and Molecular Biology, University of Sevilla School of Medicine, 41004, Sevilla, Spain; 4 Department of Physiology (Cell Physiology Research Group), University of Extremadura, 10003, Cáceres, Spain

**Keywords:** *Clostridium perfringens*, Immune response to vaccination, Melatonine, Platelet aggregation, Pregnant sheep

## Abstract

**Background:**

Melatonin regulates several physiological processes and its powerful action as antioxidant has been widely reported. Melatonin acts modulating the immune system, showing a protective effect on the cardiovascular system and improving vaccine administration as an adjuvant-like agent. Here, we have investigated the role of melatonin as an adjuvant of the *Clostridium perfringens* vaccine in prepartum sheep and whether melatonin modulates platelet physiology during peripartum.

**Results:**

The experiments were carried out in peripartum sheep from a farm located in an area of Mediterranean-type ecosystem. Plasma melatonin levels were determined by ELISA and sheep platelet aggregation was monitored using an aggregometer. Here we demonstrated for the first time that plasma melatonin concentration were higher in pregnant (125 pg/mL) than in non-pregnant sheep (15 pg/mL; *P < 0.05*). Administration of melatonin prepartum did not significantly modify platelet function but significantly improved the immune response to vaccination against *C. perfringens*.

**Conclusion:**

Administration of melatonin as an adjuvant provides a significant improvement in the immune response to vaccine administration prepartum against *C. perfringens*.

## Background

Melatonin (N-acetyl-5-methoxy-tryptamine), a hormone produced in the pineal gland and a number of other cells and organs, regulates a number of physiological processes either by its powerful ability to scavenge reactive oxygen species, interaction with intracellular molecules or via activation of the G protein-coupled melatonin receptors, MT1 and MT2 [1-3]. Melatonin regulates the immune system by affecting cytokine production, enhancing the production of several T helper cytokines and modulating the IL-2/IL-2R system [4-6]. Melatonin has been shown to exert a positive effect as an adjuvant of a number of vaccines [7-11]. Platelet function is also regulated by melatonin binding to high-affinity sites [12], which results in enhanced platelet responsiveness to physiological agonist and inducing beneficial effects on platelet function and haemostasis [8].

Enterotoxemia, caused by *Clostridium perfringens,* is one of the most frequently occurring diseases in all species of domestic animals and humans inducing gastrointestinal and enterotoxemic diseases in animals [13-15]. The bacterium is a normal inhabitant of the intestine where it produces small amounts of toxin that, under normal conditions, are removed by gut movements or are inactivated by circulating antibodies. Prepartum vaccination of sheep has been recommended due to the significant increase in lamb antibody concentration compared to lambs reared by unvaccinated sheep [16]. In addition, this manoeuvre has been found to reduce the frequency of fecal shedding of serogroup C1 salmonellae during the peripartum period [17]. Here we have investigated the effect of melatonin administration as an adjuvant of the *C. perfringens* vaccine administered prepartum on the immune response. In addition, we have explored whether vaccination against *C. perfringens* alters platelet function, as shown for the vaccine against *Dichelobacter nodosus*[8].

## Results and discussion

Rectal temperature, as well as the observation of possible local or general adverse reactions, is important for the evaluation of the innocuousness of vaccine or melatonin administration. We have not found any local or general clinical signs, including granulomas, after administration of the vaccine or subcutaneous implants of melatonin in our experimental groups. In addition, the rectal temperature was in the physiological range.

Serum concentration of melatonin in the different experimental groups is shown in Table 1. In control animals (not vaccinated, not treated with melatonin), the plasma level of melatonin was quite stable with no significant changes during the performance of the experiment. Vaccination *per se* had no significant effect on the serum level of melatonin. As expected administration of melatonin by implant induced a significant increase in the serum concentration of melatonin, which was maintained elevated for 60 days and then returned to basal levels in animals with melatonin implant.

**Table 1:** Plasma melatonin concentration (pg/mL) at the time of the vaccination procedure at 10:00 A.M

**Treatment**			**Day**		
	**0**	**21**	**42**	**60**	**90**
I. Control	123 ± 10	132 ± 12	127 ± 12	131 ± 8	126 ± 10
II. Melatonin Implant	125 ± 10	277 ± 14^*†^	296 ± 15^*†^	292 ± 12^*†^	261 ± 10
III. Vaccine	120 ± 14	126 ± 10	124 ± 12	128 ± 12	130 ± 10
IV. Vaccine+Melatonin Implant	126 ± 12	295 ± 11^*†^	328 ± 13^*†^	313 ± 9^*†^	287 ± 14^*†^

Values represent mean melatonin concentration as determined by enzyme-linked immunosorbent assay (ELISA). Data are expressed as pg/mL and presented as mean ± S.E.M. of 10 experiments. ^*^P < 0.05 compared with day 0. ^†^*P < 0.05* compared with their respective control in non-vaccinated animals.

Melatonin plays a role in synchronizing the reproductive responses of animals to environmental light conditions; it has been reported that serum melatonin levels during human pregnancy are higher than in a non-pregnant state [18]. Here, we showed for the first time in sheep, the differences in plasma melatonin concentration between non-pregnant and pregnant sheep. As we showed previously, sheep in non-gestational state have an average plasma melatonin concentration of 15 pg/mL [8], considerably smaller than pregnant sheep (125 pg/mL) (Table 1). Differences in plasma melatonin concentration may be due to strong melatonin antioxidant properties, as well as, as previously postulated, high levels of melatonin during pregnancy may be one of the factors that reduce oxidative damage from ROS in the placenta and systemic endothelial cells [18-21].

Plasma antibody concentration in the different experimental groups is shown in Table 2. As expected, in control subjects and those treated by melatonin implants the plasma antibody concentration was unaltered during the experiment, while vaccine administration increased significantly plasma antibody concentration reaching the highest level after 60 days of vaccination (Table 2; *P < 0.05*). As shown in Table 2, in animals treated with melatonin implants the concentration of plasma antibodies was significantly greater after vaccination, which further support a positive role of melatonin in antibody production upon vaccination, as described in non-pregnant sheep [10]. Interestingly, while the effect of melatonin on the serum antibody level after vaccination of non-pregnant sheep was found to be slightly greater but not statistically different from the levels in vaccinated animals in the absence of melatonin [10], when the vaccination takes place prepartum, the serum antibody concentration was significantly greater in vaccinated controls in the presence of melatonin; thus suggesting that the time of vaccination plays a important role in the effect of melatonin on the immune response, being more effective when administered prepartum.

**Table 2:** Serum antibody levels at the different treatments

**Treatment**			**Day**		
	**0**	**21**	**42**	**60**	**90**
I. Control	13 ± 2	12 ± 3	14 ± 3	16 ± 4	15 ± 3
II. Melatonin Implant	12 ± 2	16 ± 3	14 ± 4	13 ± 3	15 ± 2
III. Vaccine	16 ± 3	40 ± 6^*†^	90 ± 5^*†^	104 ± 4^*†^	70 ± 5^*†^
IV. Vaccine + Melatonin Implant	14 ± 3	52 ± 5^*†§†^	112 ± 6^*†§†^	141 ± 5^*†§†^	97 ± 4^*†§†^

Values represent mean antibodies levels as determined by microagglutination assays. Data are presented as mean ± S.E.M. of 10 experiments. ^*^P < 0.05 compared with day 0. ^†^*P < 0.05* compared with their respective control in nonvaccinated animals. ^§^*P < 0.05* compared with the application of melatonin implants. ^†^*P < 0.05* compared with vaccinated animals.

Melatonin has also been reported to induce a number of immune responses besides antibody production, including enhance antigen presentation to the immunocompetent cells, as described in mice, where melatonin improved antigen presentation by macrophages, by increasing the expression of MHC class II and stimulated activation of T helper cells [22,23]. Furthermore, melatonin may modulate the production of cytokines such as IL-2, INF-γ and IL-6 and increasing production IL-12 by monocytes as demonstrated in cultured human mononuclear cells [24], thus promoting Th1 cell response. In addition, melatonin may also bind to high affinity receptors present on Th2 lymphocytes from human bone marrow thus increasing the levels of IL-4 [25], increasing the production of IL-10 and decreasing of TFN-α in mice sensitized with ovalbumin injected with complete Freund’s adjuvant, activating the Th2 response [26].

**Figure 1:**
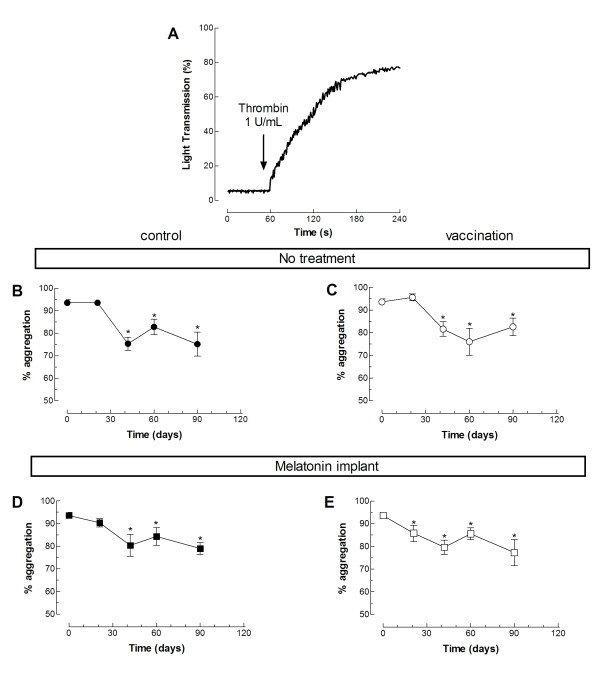
**Effect of melatonin administration on the percentage of thrombin-induced platelet aggregation in vaccinated and non-vaccinated sheep.** Platelets from sheep vaccinated or not against *Clostridium perfringens type D* and treated with melatonin or the vehicle, as indicated, were stimulated in a medium containing 1 mM Ca^2+^ with 1 U/mL thrombin. Data are mean ± S.E.M. of 10–12 independent experiments. *P < 0.05 compared with their respective controls.

**Figure 2:**
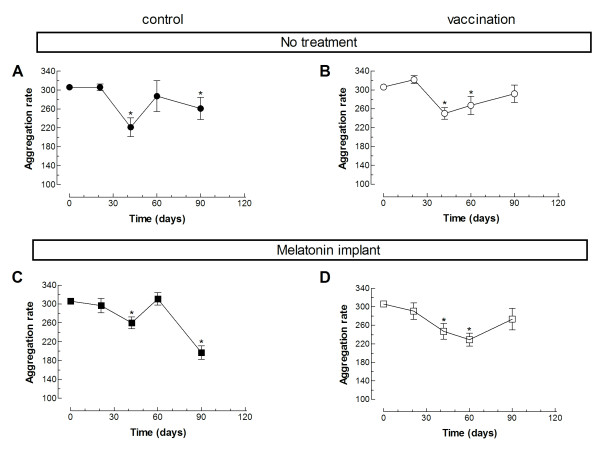
**Effect of melatonin administration in the rate of thrombin-induced platelet aggregation in vaccinated and non-vaccinated sheep.** Platelets from sheep vaccinated or not against *Clostridium perfringens type D* and treated with melatonin or the vehicle, as indicated, were stimulated in a medium containing 1 mm Ca^2+^ with 1 U/mL thrombin. Data are mean ± S.E.M. of 10–12 independent experiments. *P < 0.05 compared with their respective controls.

**Figure 3:**
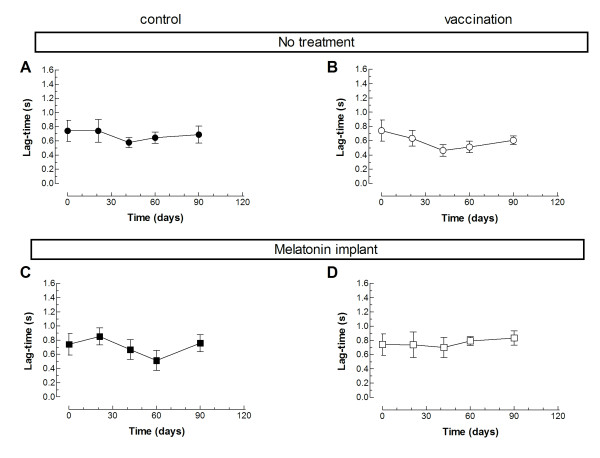
**Effect of melatonin administration in the lag-time of thrombin-induced platelet aggregation in vaccinated and non-vaccinated sheep.** Platelets from sheep vaccinated or not against *Clostridium perfringens type D* and treated with melatonin or the vehicle, as indicated, were stimulated in a medium containing 1 mm Ca^2+^ with 1 U/mL thrombin. Data are mean±S.E.M. of 10–12 independent experiments.

We have further investigated whether vaccination against *C. Perfringens* (D serotype) in the absence or presence of melatonin might have any effect on platelet function, and thus, hemostasis. Hence, we tested *ex vivo* platelet aggregation in response to the physiological agonist thrombin in the four experimental groups. The percentage of aggregation upon stimulation with 0.5 U/mL thrombin decreased significantly after partum in all the experimental conditions (Figure 1; *P* < 0.05; n = 10–12). Similar results were obtained when we calculated the rate of aggregation (Figure 2; *P* < 0.05; n = 10–12). Interestingly, the lag-time in response to thrombin was not altered in any of the groups investigated (Figure 3). Vaccination or treatment with melatonin did not modify agonist-induced aggregation in sheep during the last stages of pregnancy and postpartum period. We have found that in pregnant sheep serum melatonin concentration is almost 10 times greater than in non-pregnant subjects [8]. Therefore, melatonin administration results in a 2- over 20-fold increase in melatonin concentration in pregnant and non-pregnant animals, respectively, where the serum melatonin concentration reached similar values after exogenous administration.

## Conclusions

We have found that pregnant sheep serum melatonin concentration is elevated as compared to non-pregnant animals. The enhanced level of melatonin in pregnant subjects may be attributed to a physiological effect to avoid oxidative disorders derived from pregnancy, where melatonin could exert an important role as antioxidant preventing platelet hyperactivation, due to its inhibitory effect over COX-1 and arachidonic acid-induced aggregation. The lack of effect of melatonin on platelet aggregation might be explained by desensitization of melatonin receptors after prolonged exposure to elevated concentrations of melatonin, as previously reported [27].

The ability of adjuvants to enhance the immune responses to vaccine antigen has long been investigated and the search for adequate adjuvants is critical for improving the performance of existing vaccines, and a role of melatonin as adjuvant has been described [7-11]. Our results indicate that melatonin significantly enhanced the immune response to vaccination against the *Clostridium perfringens type D*, which, together with our previous studies in non-pregnant sheep, strongly suggest that melatonin enhances the immune response to vaccination. In addition to our previous findings, here we demonstrate for the first time the beneficial effects of melatonin on the immune response to vaccination when administered prepartum.

## Methods

### Materials

Apyrase (grade VII), EGTA, aspirin, bovine serum albumin (BSA), melatonin and thrombin were from Sigma–Aldrich 1(Madrid, Spain). Calcein and fura-2/AM were from Molecular Probes (Leiden, The Netherlands). Melatonin ELISA was from IBL (Hamburg, Germany). All other reagents were purchased from Panreac (Barcelona, Spain).

### Experimental set-up and animal immunizations

The experiments were carried out in a commercial farm located in an area of Mediterranean type ecosystem in Cáceres (Spain) over a 8-months period (October to May, 2009–2010). A total of 40 pregnant sheep (30 months old; average weight 47.64 Kg) of the Merino breed and meat producing were used. Animals were kept without restraint in their habitual herd and were grown up in an extensive breeding system accordingly with regional and European Union standard regulations.

The study was approved by the Ethical Committee of the University of Extremadura (Cáceres, Spain) in accordance with the National Institute of Health Guide for the Care and Use of Laboratory Animals.

The animals were divided into four different experimental groups; each one comprised of 10 female sheep. Formulation of the immune-preparations, dosage, administration routes as well as different tests carried out for each animal are summarized (Table 3). Animals were treated 10 days before the onset of the experiment with Ivermectine subcutaneously a dose of 1 ml per animal (Ivomec^TM^, Merial Laboratories S.A., Barcelona, Spain) to deworm them. Eight days later first blood samples from the 40 animals were drawn to check blood levels of melatonin and platelet function. Blood samples and sera preparation were performed according to standard procedures from external jugular vein at the same time (10.00 A.M.).

**Table 3:** Group composition and chronological schedule of tasks carried out in each animal

**Group**	**Composition**		**Dose**	**Administration**	
**I**	Mock control				
**II**	Control+Melatonin implant				
**III**	Vaccine		2 mL	subcutaneus	
**IV**	Vaccine+Melatonin implant		2 mL	subcutaneus	
**Day**	**0**	**21**	**42**	**60**	**90**
Vaccination	+	+			
Melatonin implant	+	+			
Blood collection	+	+	+	+	+
Platelet function	+	+	+	+	+
Melatonin measurement	+	+	+	+	+
Rectal temperature	+	+			
Local reaction	+	+			
General reaction	+	+			

Sheep were subjected, on deworming day, to estrus synchronization protocol, involving intravaginal sponge implantation containing flugestone acetate (Chronogest 20 mg, Intervet Laboratories, Madrid, Spain). 14 days after synchronization, we inoculated 50 UI of PMSG (Foligon, Intervet Laboratories, Madrid, Spain) via intramuscular injection to each subjects. Natural mating took place 55 hours after application of PMSG and all the 40 sheep became pregnant. Animal gestation state was corroborated by performing trans-abdominal ultrasound using a 5 MHz linear probe (HS-1500 V, Honda Electronics Co., LTD., Japan). Day 130 of gestation was considered as the day 0 (D 0) of the study. Vaccination of the 20 female sheep, corresponding to groups III and IV (Table 3), was performed subcutaneously in the area of the axilla with a dose of 2 mL of the vaccine. The immune-preparation consisted of *C. Perfringens type D* (Ovejero Laboratories, Leon, Spain). Administration of the booster vaccine injection was carried out at D. 21. To assess the overall response to the vaccination procedure, some basic clinical signals were monitored (Table 3). Rectal temperature was recorded during 3 consecutive days after first vaccination and re-vaccination. Rectal temperature was always monitored at the same time (10.00 A.M.), to avoid fluctuations due to defferent metabolic conditions. We also assessed the general and local adverse reactions by clinical and macroscopic observation, to detect any reaction due to the vaccination.

The clinical observation of the animals the day of first and booster vaccination and three days after vaccination did not show granulomas or other relevant signs at the administration sites. The rectal temperature of the animals included in the experiments was homogeneous and clinically normal after the administration of the vaccine or melatonin, ranging from 38.7 to 39.5 °C.

### Melatonin treatment

On day 0 and 21, female sheep from groups II and IV received slow release 36 mg/animal subcutaneous implants of melatonin in the left ear (Melovine®, Ceva Sante Animale, France).

### Platelet preparation

Blood was obtained from sheep, and mixed with one-sixth volume of acid/citrate dextrose anticoagulant containing (in mm): 85 sodium citrate, 78 citric acid and 111 d-glucose [28,29]. Platelet-rich plasma was then prepared by centrifugation for 5 min at 700 g and aspirin (100 μm) and apyrase (40 *μ*g/mL) were added. Cells were collected by centrifugation at 350 g for 20 min and resuspended in HEPES-buffered saline (HBS) containing (in mm): 145 NaCl, 10 HEPES, 10 d-glucose, 5 KCl, 1 MgSO4, pH 7.45 and supplemented with 0.1% w/v BSA and 40 *μ*g/mL apyrase.

### Cell viability

Cell viability was assessed using calcein and trypan blue. For calcein loading, cells were incubated for 30 min with 5 lm calcein-AM at 37 °C, centrifuged and the pellet was resuspended in fresh HBS. Cells were treated with the different inhibitors, centrifuged and resuspended in HBS. Fluorescence was recorded from 2 mL aliquots using a spectrophotometer (Varian Ltd, Madrid, Spain). Samples were excited at 494 nm and the resulting fluorescence was measured at 535 nm. The results obtained with calcein were confirmed using the trypan blue exclusion technique. About 95% of cells were viable in our platelet suspensions and no effect was observed after sheep treatment with melatonin.

### Platelet aggregation

The percentage, rate and lag-time of aggregation in washed platelets were monitored using a Chronolog aggregometer (Havertown, PA, USA) at 37 °C under stirring at 1200 rpm [30]. The percentage of aggregation or amplitude is estimated as the percentage of the difference in light transmission between the platelet suspension in HBS and HBS alone and indicates the percentage of platelets that aggregate in response to an agonist. Resting platelets in suspension are arbitrarily considered by the aggregometer as 0% aggregation and HBS is considered to be 100% aggregation. The rate, or slope, of the aggregation is the percentage change of aggregation per minute. The lag-time is the time between platelet stimulation and the initiation of aggregation. Percentage of aggregation, aggregation rate and lag-time were calculated using AGGRO/LINK®. As previously reported [28], treatment of platelets, suspended in a medium containing 1 mM Ca^2+^, with 1 U/mL thrombin induced rapid aggregation characterized by a large increase in light transmission as the platelets aggregated (Figure 1; n = 10)A.

### Measurement of melatonin in serum

Blood samples (10 mL/animal) were collected at the same time (10.00 a.m.) by syringe and transferred to a tube containing serum-separating gel. Samples were centrifuged at room temperature for 15 min at 300 *g*. Serum was then divided into aliquots in Eppendorf vials and kept frozen at −30 °C until the time of assay.

Melatonin levels were determined by a Melatonin ELISA (Immuno Biological Laboratories, Hamburg, Germany) according to the manufacturer’s instructions. Melatonin from 0,5 mL of the samples, standards and controls was extracted (90–100% yield recovery) using C18 reversed phase columns (Immuno Biological Laboratories) and methanol elution. The dried extracts (after evaporating methanol) were stored at −20 °C for up to 48 h. Melatonin levels were measured in duplicate using 96-well microtiter plates coated with captured antibody goat anti-rabbit Ig. Each microtiter plate was filled either with 50 mL blank reagent, extracted calibrators, extracted samples or extracted standard solutions (containing 0, 3, 10, 30, 100, or 300 pg/ml of melatonin). Then, 50 mL of melatonin biotin and 50 mL of rabbit-antiserum were added into each well, shaken carefully, sealed with adhesive foil and incubated overnight (14–20 h) at 2–8 °C. After washing three times with 0,25 mL diluted assay buffer, 0,15 mL of anti-biotin conjugate to alkaline phosphatase was added to each well and incubated for 2 h at room temperature. The reaction was developed using p-nitrophenyl phosphate and optical densities were determined at 450 nm in an automatic microplate reader.

The sensitivity of the MLT assay was 3 pg/mL. Both the intra and inter-assay coefficients of variation (CV) were <10%. For simplicity, the results are expressed as the respective means in pg/mL of the serum melatonin levels.

### Measurement the titer of antibodies in serum

The titer of antibodies was determined by microagglutination assays. Briefly, serial dilutions of the sera up to 1/10240 in phosphate buffer saline O (PBS-SO) were applied using 96 V bottom plates (Soria Greiner, Spain). Then, 50 μL of antigen suspensions were deposited in each well. The plates were incubated at 37 °C for 12 h and then refrigerated at 4 °C for 2 h before we proceeded to read them. The appearance of a button at the bottom of the bowls, due to precipitation of free antigen was considered a negative result. If, on the contrary, a coloured diffusion of the contents appears at the bottom of the bowl, it means agglutination occurred (antigen-antibody complex) and the result was considered positive.

### Statistical analysis

Analysis of statistical significance was performed using Student’s *t*-test, Two-way Anova and Bonferroni posttests. *P <* 0.05 was considered to be significant.

## Competing interests

The authors declare that they have no competing interests.

## Authors’ contributions

IJ and JAR participated in the study design, the experimental work, the analysis interpretation of the data and drafted the manuscript. SR, AR and MPM participated in the study design and the experimental work. ACV participated in the experimental work. All authors read and approved the final manuscript.

## Authors’ information

This work was supported by Junta de Extremadura-FEDER (PDT08A020 and GR10010) and by MICINN grant BFU2010-21043-C02-01. IJ was supported by a MICINN grant BES-2008-002875. We thank the Ovejero Laboratoires (Spain) for kindly providing the immune-preparation of *Clostridium Perfringens type D.*
